# Promoting occupational health interventions in early return to work by implementing financial subsidies: a Swedish case study

**DOI:** 10.1186/1471-2458-13-310

**Published:** 2013-04-08

**Authors:** Christian Ståhl, Allan Toomingas, Carl Åborg, Kerstin Ekberg, Katarina Kjellberg

**Affiliations:** 1 National Centre for Work and Rehabilitation, Department of Medical and Health Sciences, Linköping University, Linköping 581 83, Sweden; 2 Karolinska Institutet, Institute of Environmental medicine, Unit of Occupational medicine, Norrbacka, Stockholm, Sweden; 3 Helix Vinn Excellence Centre, Linköping University, Linköping 581 83, Sweden

**Keywords:** Occupational health, Return to work, Implementation, Sweden, Subsidies, Employers

## Abstract

**Background:**

In 2010, the Swedish government introduced a system of subsidies for occupational health (OH) service interventions, as a part in a general policy promoting early return to work. The aim of this study was to analyse the implementation of these subsidies, regarding how they were used and perceived.

**Methods:**

The study was carried out using a mixed-methods approach, and comprises material from six sub-studies: a register study of the use of the subsidies, one survey to OH service providers, one survey to employers, one document analysis of the documentation from interventions, interviews with stakeholders, and case interviews with actors involved in coordinated interventions.

**Results:**

The subsidized services were generally perceived as positive but were modestly used. The most extensive subsidy – for coordinated interventions – was rarely used. Employers and OH service providers reported few or no effects on services and contracts. OH service providers explained the modest use in terms of already having less bureaucratic routines in place, where applying for subsidies would involve additional costs. Information about the subsidies was primarily communicated to OH service providers, while employers were not informed.

**Conclusions:**

The study highlights the complexity of promoting interventions through financial incentives, since their implementation requires that they are perceived by the stakeholders involved as purposeful, manageable and cost-effective. There are inherent political challenges in influencing stakeholders who act on a free market, in that the impact of policies may be limited, unless they are enforced by law.

## Background

A large number of studies have emphasized the need for workplace-based interventions for promoting early return to work (RTW) [1-7]. The communication between employees, employers and healthcare has been highlighted as especially important for designing adequate RTW arrangements [8,9]. There are several structural prerequisites that determine whether employers can or do engage in RTW. One central factor is the employers’ responsibilities and financial incentives for taking active measures, and this varies from one country to another [10]. For instance, Swedish employers finance the first two weeks of an individual’s sick leave, while Dutch employers finance two years; Finnish employers contribute to financing disability pensions, and workers’ compensation systems in North America are largely based on employer premiums. In many countries, employers do not have any formal responsibility for the RTW process [11].

Another factor that may influence RTW processes is the availability of occupational health (OH) services. Using OH services in rehabilitation may promote early RTW through OH service providers’ knowledge of and connection with workplaces [12,13]. While workplace-based interventions in RTW have been studied quite extensively, the use and role of OH services in such interventions has received less attention. Access to OH services differs from one country to another. In some countries (e.g. Denmark), occupational health aimed at RTW barely exists. In Finland, it is obligatory by law for employers to have an OH affiliation and the OH sector is supported by state funds, although the use of OH services is primarily financed by the labour market actors. In the Netherlands, all sick-listed persons are required to visit occupational physicians (OP) for rehabilitation purposes [14,15]. In the USA, OH is typically used either in connection with workers’ compensation laws to provide medical services for workers with occupational injuries or illnesses, or to provide general medical services aimed at workplace safety and overall worker health [16]. In Sweden and Norway, legislation states that OH services should be available when required by the working conditions. In Swedish practice, access to OH services is based on voluntary contracts between employers and OH service providers. Today, about 65% of the working population in Sweden state that they have access to OH services [17]. Small employers, however, use OH services less than larger employers [13]. OH service providers are generally not key actors in Swedish sick-listing practice: a recent study showed that most sickness certificates are issued by physicians in primary healthcare and hospitals, while only 5% of sickness certificates were issued by occupational physicians (OP) [18]. Nevertheless, OH service professionals may be involved in the rehabilitation process if the employer chooses to consult them.

### An attempt to promote OH service interventions

The number of sick-listed individuals in Sweden started to increase at the end of the 1990s, as did the duration of sickness absence spells (in particular those lasting for more than a year), reaching a peak in 2002 [19]. This development caused the government to introduce several changes to the sickness insurance systems in order to standardize the insurance process and promote early RTW. In 2008, a fixed time schedule for work ability assessments was introduced, which are to be made in increasingly broader terms as time passes (from present work tasks to the labour market at large), which affects eligibility for sickness benefits. An end-point to sickness benefits was also introduced (364 days with full benefits, plus an additional maximum of 550 days with reduced benefits after a renewed application). Further, a system of temporary disability pension for people with more long-term diseases was abolished to direct the sickness insurance system more towards labour market reintegration [20].

Following these changes, attention was also directed towards the OH service sector as a possible facilitator of early RTW. Since OH in Sweden is fully financed by employers, whereas sickness benefits are paid by the Swedish Social Insurance Agency (SSIA), there have been few incentives for employers to consult the OH services on RTW issues. Traditionally, Swedish OHS providers are consulted by employers to deliver services focused on health promotive or sickness preventive measures, and on rehabilitation after long-lasting sickness absence. However, they have generally delivered few services early in the sick-leave process. OH service providers usually know more about the working conditions of an individual worker (both on an individual and organizational level) compared with primary healthcare or hospital units, who provide most of the sickness certificates. It was argued that OH service could provide stakeholders (the employee, the employer and the SSIA) with advice that would facilitate early RTW, if introduced early after sick-listing.

In 2010, the Swedish government introduced a new financial support system to promote more extensive use of OH services in RTW. According to this, OH service providers could apply for subsidies for certain interventions, targeted at early RTW. The primary aim of the subsidies was to speed up RTW and reduce sickness absence. These interventions were thus targeted at the rehabilitation of sick-listed workers, rather than health promotion in the workplace.

There were primarily three actors involved in the formation of the subsidies: the Ministry of Health and Social Affairs, the Swedish OH service association, and the SSIA. The subsidies were initiated by the ministry, and they were developed in cooperation with the Swedish OH service association, which includes most Swedish OH service providers as members. The SSIA was involved as the authority to administer the subsidies.

Four different subsidies were introduced (sums in brackets indicate available funding for 2010; 1 € was calculated as 10 SEK):

1. A basic subsidy in relation to the number of employees connected to the OH service provider, maximum 20 € per employee and year. (10 million €)

2. A subsidy for costs of medical services, e.g. blood tests, X-rays, maximum 10 € per employee and year. (10 million €)

3. A subsidy for visits to OPs, involving a work ability assessment, 35 € per visit. (10 million €)

4. A subsidy for coordinated early interventions, involving multi-professional assessments and actions within 45 days of sick leave. OH service providers should assess the individual’s work ability, visit the workplace, assess the need for workplace adjustments, and initiate proper actions in cooperation with relevant stakeholders (including the employee and the employer), in order to promote RTW. A coordinated intervention was subsidized with 560 €. (25 million €)

The subsidies could only be granted to OH service providers who had been approved by the SSIA. To gain approval, the OH service provider should have access to competencies within work organization, behavioural science, ergonomics, health science, medical science, technology and rehabilitation. Moreover, the provider should have knowledge about the working conditions at the employees’ workplaces. The subsidies were not primarily intended to be used for health promotion, health controls or other activities not related to a specific sick-listed patient (except the basic subsidy). OH service providers should document all interventions (except for the basic subsidy) and send them to the SSIA for approval, after which the money was to be paid out to the providers. Costs for interventions were meant to be shared between employers and the OH service providers, where the providers were free to choose how to arrange this in their agreements with the employers (e.g. through reduced price for services).

### Aim

The aim of the present study was to analyse how a political attempt towards promoting OH service interventions through financial subsidies was implemented. The following research questions were asked:

1. How were the subsidies used during the first year?

2. How were the subsidies perceived by the users?

Besides a description of how the subsidies were used, the results from the study are then discussed from an implementation theoretical perspective, combining concepts from literature on implementation in healthcare settings [21] and implementation of public policies [22].

## Methods

**Figure 1:**
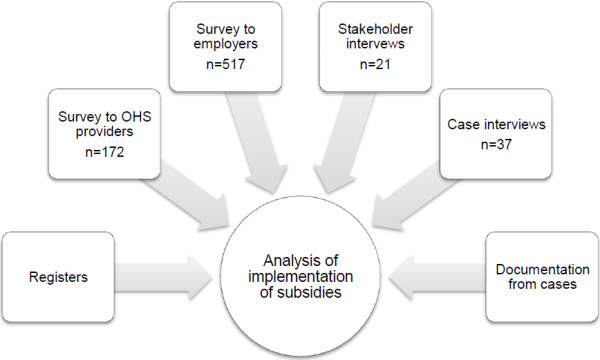
Data material that informed the analysis.

The study was carried out using a mixed-methods approach, and comprises material from six sub-studies, as shown in Figure 1. All data were collected between March and November 2011. The material from the sub-studies was analysed separately and was then combined into a joint analysis of the implementation of the subsidies. The discussion based on implementation literature thus considers all results as forming one case.

### Register study

Registers were studied to determine the use of the subsidies. The registers were obtained from the SSIA, and contained information about the amount paid out to OH service providers from the four subsidies during 2010. Data about the amount of paid subsidies of each of the four categories during 2010 to the OH service providers were retrieved from the national SSIA register. The register only contained information about whether applications were granted or not and the sums paid out, while other information given in the documents (e.g. whether workplaces were visited) had not been filed.

### Survey to employers

The employer survey contained questions about use of subsidies, and more general questions regarding use of OH services. The sampling frame was all employers in Sweden, based on a national database held by Statistics Sweden covering private, public and non-profit employers. From this database, 2000 employers where randomly chosen based on geographical variation, size, and type of business. Of the 2000 questionnaires sent out, 28 were returned to sender with address unknown. Of the 1972 remaining questionnaires, 517 of were returned (375 on paper, 142 over the web), giving a response rate of 26%. It has been estimated (although with high uncertainty) that about 65% of Swedish employers are connected to an OH service provider [17]. Under this assumption, a rough estimate is that about 40% of the target population (employers with OH service affiliations) returned the questionnaire. The survey was analysed using descriptive statistics.

### Survey to OH service providers

The OH service provider survey contained questions about which subsidies were applied for, how they were used, reasons for not applying for subsidies if not used, and what changes the subsidies had resulted in. The questions were concerned with subsidies applied for in connection with activities performed during 2010. The subsidy for coordinated early interventions was in particular focus, since it was the most comprehensive subsidy and had particular relevance for early interventions at the workplace.

The web-based questionnaire was sent by e-mail to all OH service units approved by the SSIA in May 2011. A register of e-mail addresses was obtained from the SSIA. As the intention was to gather information about how local OH service units worked with interventions financed by the subsidies, the large OH service providers in Sweden were contacted to obtain addresses to their local OH service units. In total, 302 questionnaires were sent out, with a response rate of 55%. To analyse the non-response the non-responders were contacted by telephone. Of these, 14 OH service providers were closed or merged into another company, which gives a response rate of 57%. The survey was analysed using descriptive statistics.

### Stakeholder interviews

Twenty-one people were interviewed about the use of the financial subsidies, comprising 11 representatives from OH service providers, 7 employers, and 3 SSIA officials. The selection of interviewees was based on inclusion criteria from a related study (focusing on OH services in relation to municipal and county council employers), where OH service providers were included if they reported in the OH service survey that they had municipal healthcare administrations and county councils as customers, and answered that they were willing to participate in interviews. Out of 45 OH service providers who fulfilled this criterion, a strategic selection of eight providers was made covering both in-house and external units, large and small units, and different regions in Sweden. Also, both units who had applied for the subsidy for coordinated early interventions, as well as those who had not, were represented. This selection was made in order to receive information about reasons for not applying for this particular subsidy. The selection of employers and SSIA officials was based on the contacts of the OH service representatives.

### Documentation from cases

To the authors’ knowledge, all applications sent in to the SSIA were granted subsidies. Documentation from all coordinated interventions was analysed using a summative content analysis [23]. The material consisted of documented cases sent in to the SSIA by OH service providers (n=452). The documents were retrieved from the SSIA. All 452 documents were analysed quantitatively (counting occurrences of reported intervention characteristics, such as workplace visits). In only half of the studied documents (n=222) was the information sufficient for a qualitative analysis of the interventions. These were analysed by randomly selecting ten cases for in-depth analysis, from which a coding scheme was developed for analysis of the remaining 212 cases. The analysis involved whether interventions were carried out by multiple professionals (physician and/or nurse, plus physiotherapist/ergonomist, psychologist/behavioural therapist, or other); whether interventions were carried out in dialogue with the employer, and whether workplace-oriented interventions were carried out.

### Case interviews

Interviews were carried out to follow up the document analysis, and included 14 cases where coordinated interventions had been carried out and subsidized. In total, 37 telephone interviews were carried out (14 people who received coordinated interventions, 10 of their managers, and 13 OH service representatives involved in the cases). The interviewees were randomly drawn from the cases in the document analysis and were located in different regions of Sweden. The majority of the respondents had suffered from mental problems, most commonly burnout or depression. The focus of the study was to analyse the types of interventions carried out, and how these were perceived. Data were analysed using qualitative content analysis [23,24].

The authors carried out different sub-studies, while the analyses were performed through joint discussions of the material as a whole. The different datasets approached the research questions from different methodological perspectives which served as a triangulation strategy. The material was organized into three overarching themes: use of, knowledge of and perceptions of the subsidies. All authors participated in analyzing the results, writing and approving the final manuscript.

The study was approved by the regional ethics boards of Linköping and Stockholm.

## Results

In this section, the results are presented from the six sub-studies under three general themes: use, knowledge and perceptions of the subsidies.

### Use of the subsidies

During the first year after the subsidies were introduced, only the basic subsidy was used up to the total sum available. This subsidy was also the only one where no actions or deliveries from the OH service provider were required, apart from a declaration of available competencies. The subsidies that required actions were used more modestly, and it is notable that only 2% of the available funds for coordinated interventions were used (Table 1).

**Table 1:** Amounts used of available subsidies during 2010 (Register study)

	**Available (million €)**	**Total used (million €)**	**% used**
Basic subsidy	10	10	100%
Medical services	10	2.6	26%
OP visit	10	5.9	59%
Coordinated interventions	25	0.5	2%

The employer survey showed that only 1 out of 10 employers reported that their OH service provider had applied for subsidies. Further, many did not answer this question, indicating that the subsidies were largely unknown by the majority of employers (see Table 2). Those employers who reported that their OH service provider had applied for subsidies were more often larger companies. Only 1-2% of the employers stated that they used OH services more after the introduction of the subsidies. The employer survey also indicated that employers’ general use of OH services primarily included health investigations, OP visits, psychological counselling, and ergonomic assessments, while it was less common to consult the OH service provider on rehabilitation, work environmental issues and organizational development.

**Table 2:** Proportion of employers whose OH service providers had applied for subsidies (Employer survey)

	**n**	**Yes**	**No**	**Don’t know**
Medical services	285	9%	28%	63%
OP visit	284	12%	27%	61%
Coordinated interventions	282	10%	29%	61%

The OH service survey showed that the majority of the OH service providers answering the questionnaire (85%) had applied for the basic subsidy for 2010 (Table 3). Six out of ten OH service providers had applied for the subsidy for medical services and two-thirds had applied for the subsidy for visits to OP involving work ability assessment. Four out of ten reported that they had applied for the subsidy for coordinated early interventions. In-house and external OH service providers had applied for the different subsidies to the same extent.

**Table 3:** Proportion of OH service providers who had applied for subsidies (OH service survey)

	**n**	**Yes**	**No**	**Don’t know**
Basic subsidy	151	85%	9%	6%
Medical services	151	61%	34%	5%
OP visit	152	67%	27%	6%
Coordinated interventions	149	40%	54%	6%

Among the OH service providers who reported that they had applied for the subsidy for coordinated early interventions, most of them had used the subsidy for an investigation of barriers and resources in relation to RTW, a functional capacity evaluation, and work-site visits. This would also be expected, since these three interventions were required by the SSIA in order to obtain the subsidy, although it seems that no applications for subsidies were rejected by the SSIA.

In an open question in the OH service survey, OH service providers explained their reasons for not applying for the coordinated interventions, which involved that they already had efficient routines for such services and saw no reason for engaging in a system where the cost for administration would outweigh the return.

We already work like this, but less bureaucratically and faster, without application forms and such to fill in. Since all competencies are in-house, we have a fast and efficient management of cases. This makes it difficult to sell in a service that takes longer, with the same results. (OH service survey)

Other explanations frequently mentioned in the OH service survey were that the administration was complicated and time-consuming, that the time limit for coordinated interventions (45 days) was too restrictive, that it took time to adjust practices and contracts to this new financial support system, that the rules concerning the subsidies were unclear, and that there was a lack of interest among the customers.

More than half of the OH service providers stated in the OH service survey that the subsidies had not affected their work routines regarding early occupational rehabilitation, while 40% believed their work routines had changed (Table 4). Changes in work routines that were mentioned involved earlier interventions in the sick-leave process, better quality, routines and structures for the rehabilitation work, increased team work, increased access to different occupational health competencies (for example by hiring new professionals), and better cooperation with the employers.

**Table 4:** Changes in OH service providers’ work with early occupational rehabilitation, after the introduction of the subsidies (OH service survey)

	**n**	**Yes**	**No**	**Don’t know**
Work routines in early occupational rehabilitation have changed	147	40%	53%	7%
The number of assignments for early interventions has increased	146	23%	66%	11%
Contracts with employers have changed	149	6%	85%	9%

Two-thirds of OH service providers thought that the number of assignments for early interventions had not increased (Table 4). Relatively few OH service providers claimed that the number of assignments had increased (23%), which was due to already established work routines for early interventions, low sickness absence, and alternative cooperation routines with employers. Also, only a few of the OH service providers reported that contracts with their customers had changed after the subsidies were introduced (6%).

The document analysis showed that when coordinated interventions were carried out, they were generally multidisciplinary and designed in dialogue with the employer (Table 5). However, it was common that the interventions were limited to assessments and investigations (e.g. work ability assessments without subsequent actions). Workplace adjustments or other work-related interventions were documented in less than half of the cases (noted as “workplace interventions” in Table 5).

**Table 5:** Proportions of cases fulfilling three quality criteria, based on documentation of coordinated interventions to the SSIA (Document analysis, n=222)

	**Yes**	**No**
Multidisciplinary interventions	81%	19%
Contact with employer	89%	11%
Workplace interventions	44%	66%

### Later use of the subsidies

Figures from the SSIA show that the sums for the subsidies have been gradually reduced since their introduction (SSIA website). The sum for the first year (2010) was 55 million €, of which 19 million € were used. In 2011, the allotted sum was reduced to 48 million €, of which 25,7 million € were used. Also in 2011, the coordinated interventions were the subsidy least used (5,4% of the allotted sum). In 2012, 35,5 million € have been allotted, which suggests that the subsidies are being gradually phased out.

### Knowledge of the subsidies

The OH service providers who were interviewed were (with a few exceptions) aware of the subsidies, although they sometimes lacked knowledge of how they were to be applied (Stakeholder interviews).

The employer survey suggests that the employers were unfamiliar with the subsidies. In the case interviews, only two out of ten managers had heard of the subsidies. In most cases, neither the people receiving interventions nor the employers were aware that the intervention was subsidized, although the manager was required to sign a form where the intervention was documented.

Interviewer: These subsidies, are you familiar with them?

Employer: No. No, I’ve no idea. (Case interviews)

Of the actors involved, employers and employees receiving interventions thus seemed least aware of the subsidies. The fact that employers had such low awareness of the subsidies suggests that they were not always given sufficient information by their OH service providers. Some OH service providers reported in interviews that the subsidies were only used with municipal employers and not marketed to other customers:

OH service: It’s the municipality that requested this, and of course the OH service spread it, because they make money from this. And another municipality has started requesting it too.

Interviewer: Have you marketed this to other types of employers?

OH service: No, we haven’t, no. (Case interviews)

This selective marketing is reflected by OH service providers’ general perceptions of different employers’ willingness to engage in RTW, where larger employers were seen as having better resources for participating in rehabilitation.

OH service: Yes, of course there are differences, since this [interest in rehabilitation] depends to a large extent on the specific individual, what interest he or she has as a manager. But also on the rehabilitation policy in the company, what culture there is. If it’s a small company it may be more difficult than for a larger one where there are routines and structures for these things. (Case interviews)

Some OH service providers also seemed to adapt a trial-and-error approach, where subsidies were used on a smaller scale before implementing them on a routine basis. For instance, providers reported how they had initiated coordinated interventions in relation to some (in this case, municipal) employers before introducing them more widely to their customers.

OH service: We have limited this to the municipal care department, as a routine, and then we will accept all departments. […] We haven’t got the resources to work in this way with all departments – this isn’t the only assignment we’ve got here. (Case interviews)

The use of OH service in rehabilitation thus seems to be most common in those cases where the employer has well-established RTW routines and connections with an OH service provider.

OH service providers had different perceptions of whether the subsidies were supposed to reduce costs for both providers and employers, or only for the provider. Some OH service providers claimed that they aimed to split the subsidy between them and the employer; some that the interventions could be provided to a reduced cost; while some did not seem to bother communicating this to the employers at all.

OH service: We never had that discussion with them [the employers]. I know others did, they talked about having seen some formulation about employers being supposed to pay half the sum, and the rest being covered by the subsidy. But we haven’t had a discussion of prices. We told them that there were various possibilities, and that there were formalities involved, and there was no more discussion about it. We’ve worked as usual and haven’t talked about these things. (Case interviews)

How (and if) the subsidies would be distributed between OH service providers and employers was thus unclear, and was not clearly communicated by the SSIA. An OH service representative mentioned how the information about this changed over time:

OH service: Before I got involved I know they [the SSIA] had information meetings, and back then everybody thought that this compensation would only go to the employer. So that turned out to be a bit wrong, since we were the ones who received the subsidies. (Case interviews)

Thus, since information about the subsidies generally did not seem to reach the employers, they did not appear to be aware that the costs for the interventions could be reduced through the subsidies.

### Perceptions of the subsidies

The OH service providers’ generally perceived the initiative of the subsidies as positive (OH service survey, stakeholder and case interviews), although often being skeptic toward the overly bureaucratic administration, especially regarding the coordinated interventions.

OH service: It’s this procedure back and forth with the subsidies that’s difficult. We’ve had several sessions about it, and we still hear “I don’t understand any of this”. That’s how we perceive it. So nobody dares to use it. (Stakeholder interviews)

The perceived usefulness of the subsidies was limited due to the complexity of how the subsidies were formulated and administrated (OH service survey, stakeholder and case interviews). A recurrent opinion was that the subsidized services would demand more resources for administration than the subsidy would cover, which was one of the explanations given for not applying.

This perceived complexity was most commonly attributed to the coordinated interventions (OH service survey, stakeholder and case interviews). Despite the fact that these interventions were perceived as the most useful subsidy for facilitating RTW, these were the ones that were used the least. When coordinated interventions were carried out, OH service providers perceived that the RTW process was faster and more purposeful. By some, the coordinated approach was perceived as being close to the OH service providers’ ideal of how a rehabilitation process should work, with all actors involved in continuous dialogue and cooperation.

Further, the scope of the subsidies was criticized: several OH service providers wanted to use subsidies also for preventive work and not only for rehabilitation (OH service survey, stakeholder interviews).

The subsidized measures were in some cases perceived as difficult to apply to existing routines in planning rehabilitation interventions, since they did not rhyme with the preventive ambitions in regular practice.

OH service: I think the subsidies should rather be used for planning interventions and more forward-looking measures, instead of tying them to sick leave. This formulation is a bit unfortunate, since it doesn’t support preventive thinking in the workplaces. (Stakeholder interviews)

Another contextual factor for the modest use was the range of customers and the types of contracts, which relates to the structural characteristics of the OH service market. Some contracts with employers were formulated in ways that made it difficult to use subsidized measures (e.g. long-term contracts with certain services), and in some, the contract already comprised the types of services that were subsidized, but where other routines for this were already settled.

## Discussion

A central finding was the limited extent to which the subsidies were used, especially the more extensive subsidy for coordinated interventions. Although the contents of the subsidized services were generally perceived as positive, most OH service providers reported no changes in work routines. Further, the subsidies were largely unknown by employers. OH service providers’ explanations for not using the subsidies point to a perceived lack of advantage and too high costs for applying for subsidized services, compared with regular practice. This is further illustrated by the extensive use of the only subsidy which did not require any actual service deliveries (the basic subsidy) while those where this was demanded were used less than expected. OH service providers also reported on a mismatch between the design of the subsidies and the priorities of the OH service providers, who often prioritized preventive interventions which was not within the scope of the subsidies.

### Why were the subsidies for coordinated interventions not used?

It is relevant to ask whether the minimal use of the coordinated interventions (the subsidy most strictly focused on RTW) were due to the interventions themselves, to the dissemination of the subsidies, or to characteristics of the users (OH service providers and employers). In answering this, it is relevant to ask whether the potential users *understood, were able* to and *wanted* to implement the interventions [25]. These questions refer to different domains identified by implementation literature.

Research on implementation has developed within different disciplines, of which the most elaborated traditions can be found in studies of evidence-based practice in healthcare settings (often labelled “implementation science”), and in studies of public policy implementation. Within the former tradition, a comprehensive conceptual framework has suggested five domains of determinants for implementation outcomes: intervention characteristics, outer setting, inner setting, characteristics of the individuals involved, and the process of implementation [21]. These domains contain a broad variety of determinants targeting individual, organizational and societal factors, and correspond well to other studies on implementation in healthcare (cf. [26-28]). In the literature on policy implementation, a range of variables has been identified for analysing implementation, and one comprehensive review presented the following seven variables: policy characteristics, policy formation, vertical public administration, responses of implementation agencies, horizontal inter-organizational relationships, responses from those affected by the policy, and environment or policy context [22]. In many respects, the two traditions have presented similar determinants for implementation studies, although with different focus. The present study analyses policy-driven interventions in an occupational health setting. Thus, the following discussion will combine concepts used in the two fields, based on which are better fitted to explain the different parts of the implementation process (where policy implementation literature is generally more sensitive to organizational and political dimensions, for instance).

The *intervention characteristics* involve the relative advantage of the intervention related to other methods, the adaptability of the intervention to local conditions, whether it is possible to try out the intervention before using it on a full scale (trialability), complexity and cost [21]. The use of the subsidies was influenced by their design: the complexity involved in applying for and administering subsidies was repeatedly mentioned as impeding the implementation. OH service providers generally perceived coordinated interventions as too complex to administer within the given time limit of 45 days, although the contents of the actual interventions were perceived as useful. In this respect, the advantage of applying for subsidies compared with regular practice was perceived as low. The costs involved were also attributed to the heavy administration. Adaptability was perceived differently: some managed to integrate the coordinated interventions into their regular routines, while others did not. Some OH service providers mentioned that they tried using the interventions on a smaller scale, and then planned to increase the use of them, indicating that the trialability of the subsidies was good.

Policy implementation literature has discussed *policy characteristics* in terms of their ambiguity (whether or not they are based on knowledge) and conflict (whether or not different stakeholders agree) [29]. The evidence base for these subsidies may be perceived as relying on the acknowledgement of workplace-based interventions and early RTW [2,5]. The conflict between stakeholders could refer to different ideas regarding the responsibilities for rehabilitation, in that the employers, the OH service providers, regular healthcare and the SSIA are all involved with different roles. The subsidies aimed to promote workplace-oriented interventions by engaging OH service providers in RTW processes, which thus was a policy ambition that may have been perceived differently by different stakeholders, due to differing perceptions on the responsibility for carrying out such interventions. The material of this study indicates that both employers and OH service providers generally agree on employers’ responsibility for workplace interventions, although the role for OH services in facilitating or participating in such interventions is not equally clear.

The *outer setting* involves policies, incentives and the overall political context in which the implementation takes place [21], which roughly corresponds to the *policy context* in the literature on policy implementation [22]. The results indicate that it was mostly large employers with well-established connections to OH service providers who used the subsidies. The problems of involving smaller employers in OH service interventions are reasonably an effect of the structural conditions for OH in the Swedish system, in that it is difficult for employers with scarce resources to afford OH services [13]. It could also be argued that the changing structure of the labour market (towards more insecure employment contracts and increased use of temporary agency workers, cf. [30]) makes OH service interventions more difficult. Studies have also pointed to the increased vulnerability of those with temporary or precarious employment contracts [31,32]. It could be assumed that the interest for employers to offer OH services would decline as the use of temporary agency workers increases. Within the Swedish system, the responsibility for rehabilitation of sick-listed temporary agency workers lies with the temporary work agency, where the decision whether to use OH services in rehabilitation (as well as the cost) is placed on the temporary work agency. The extent of OH service use in such settings is yet to be studied.

In a system where employers’ use of OH services is optional and provided on a free market, it is difficult for governments to promote the use of specific services. If it is not possible for the authorities to implement OH service interventions through legislated employer responsibilities or mandatory OH service consultations, promotional activities will need to be based on various forms of incentives. Financial incentives may take different forms. In many workers’ compensation systems, employers finance the system by paying experience-rated premiums, which has been shown to increase employers’ claims and cost management activities [33]. Other studies have shown that subsidized wages for disabled people (if sufficiently generous) may be effective for making employers more positive to employ disabled people, although risking to create a segregated form of employment for people with disabilities [34]. The financial incentives in this study, however, were mostly targeted towards OH service providers, with the implicit intention that employers would be offered cheaper OH services. The study illustrates how such intentions were not realized, due to the problems in influencing the relationships between actors on a market in which governmental regulation is low.

Governments may introduce either permissive or enforcing regulations, where the former will be followed primarily if they are perceived as corresponding to the actors’ own goals, interests and values, and it is common that actors fail to follow rules since they simply do not know about them [35]. Financial subsidies is an example of a set of permissive rules, where the use was determined by whether the OH service providers and the employers knew about them, and whether they were perceived as applicable and useful (cf. [25]). As reported in the results, both the knowledge of the subsidies and the perceived usefulness can be questioned, which serves as one explanation for the low use. A more enforcing regulation could possibly have implied that the regulation would have been better known and applied more broadly. However, introducing such regulations would mean that the system for OH services would need to change fundamentally by introducing obligations for employers, which would be a more drastic political move than introducing a subsidy system.

The *inner setting* involves whether an organization has a capacity for change, how work is organized, and the networks between organizations [21]. Policy implementation here discusses *implementation agencies’ responses* and *inter-organizational relationships*[22]. The OH service providers approached the subsidies differently: some adapted their organizations more than others in order to integrate the subsidized interventions into their routines. The relationships between OH service providers and employers also differed: some OH service providers seemed to inform employers and discuss the use of the subsidies, while others seemed to prefer to leave the employers out of the process. The results suggest that those OH service providers who managed to integrate the interventions into their regular routines were satisfied with the content of the interventions, although they were still critical towards the administration required.

*Characteristics of individuals* involves aspects such as knowledge and beliefs regarding the intervention [21], which varied between OH service providers, and between professionals. Employers also varied in their knowledge of the subsidies: most did not know about them, while others were engaged through tight connections with OH service professionals, especially where there was an in-house OH service provider. Generally, the implementation process is facilitated if interested and committed people (described as “champions” in the literature [21]) are involved. This could be observed in interviews with OH service representatives. Individual responses to the subsidies thus differed with regard to how they understood the policy, and how they responded to it [22].

The *process of implementation* focuses on the activities carried out to implement the intervention, such as planning, engaging stakeholders, informing users, and evaluating results [21]. Policy implementation literature also focuses on how policies are *formed*, and how this is communicated *vertically*, to the street level [22], and on the impact of professionals executing the policies, who by some are considered as being policy-makers in their own right [36]. In this study, the results point at a rather single-handed dissemination strategy, where OH service providers were informed via the SSIA (the authority responsible for administering the subsidies) and the Swedish OH service association, while it seemed to be left to the OH service providers to inform employers. The results suggest that employers were rarely informed or engaged. Applying for subsidies and carrying out interventions was seen primarily as a concern for OH service providers, although some OH service providers had understood the subsidies as also aiming to subsidize the costs for the employer (either by splitting the subsidies between OH service providers and employers, or by giving rebates on the interventions). This reflects an ambiguity in how the subsidies were communicated from the responsible authority, where different OH service providers understood this differently. Cost reduction for employers, it seems, rarely happened.

The extensive critique of the design of the subsidies suggests that the final formulation of the subsidies did not manage to convey the needs of the users, despite the representation from the Swedish OH service association in the policy formation process.

### Summing up

The minimal use of the subsidies for coordinated interventions cannot solely be attributed to the interventions themselves, but is more likely to involve an interplay between the complexity of the application process, the costs involved, OH service providers’ organizational capacity for integrating the interventions into practice, organizational incentives and rewards related to performing the subsidized services, dissemination strategies, and legislation in the field. The implementation of the subsidies was based on a top-down strategy, where the results show how OH service providers appreciated the initiative, but largely dismissed how the subsidies were finally designed. Top-down and information-based dissemination strategies have been regarded as non-effective methods for implementation [28]; in this study, such a strategy seems to have resulted in little use of interventions that the users generally considered purposeful.

The role of workplace interventions for promoting RTW has been emphasized in several studies [1,2,4-6], and the use of OH services has been specifically pointed out as beneficial [3,12,13], although the structure for such services varies greatly between countries. The present study adds an emphasis on the complexity of involving such services in employers’ RTW routines, especially within a market-based system where their involvement is based on voluntary contracts with employers. It is reasonable to assume that prerequisites for promoting OH service interventions in RTW are better in systems where occupational health is built into the routines of sick-listing and rehabilitation processes, rather than based on optional commitments. Such differences in structural prerequisites could be targeted in future studies.

### Methodological considerations

The strength of this study lies in the breadth of the material, collected using a variety of methods. This triangulation of sources and methods adds to the validity and the reliability of the results. The low response rate in the employer survey can primarily be explained by two factors: that many employers who received questionnaires did not have access to OH services, and that the selection of respondents was too broad (including, for instance, non-profit organizations). Some workplaces that received questionnaires were also parts of larger organizations and did not have specific responsibility for OH service contracts. It is possible that the low response rate may have biased the results in different ways, in that it is likely that those respondents who were knowledgeable about the subsidies and had opinions about them were more likely to answer. The OH service survey covered all major OH service providers in Sweden, including both large and small providers from all parts of the country, and both in-house and external units. Non-responders were contacted by telephone; of these, 14 OH service providers had been closed or merged into another OH service company. In the stakeholder interviews, all employers represented municipalities or county councils, which may have biased the answers; however, case interviews involved employers from both public and private employers. In the case interviews, the little variation in types of diagnoses may be a cause of bias by not including cases with other conditions.

## Conclusions

Subsidized OH service interventions were generally perceived as positive but were used modestly. The most extensive subsidy – for coordinated interventions – was rarely used at all. Employers and OH service providers reported few or no effects on services and contracts. OH service providers explained the modest use in terms of already having less bureaucratic routines in place, where applying for subsidies would involve additional costs. Information about the subsidies was primarily communicated to OH service providers, while employers were not informed. The study highlights the complexity of promoting interventions through financial incentives, since their implementation requires that they are perceived by the stakeholders involved as purposeful, manageable and cost-effective. There are inherent political challenges in influencing stakeholders who act on a free market, in that the impact of policies may be limited, unless they are enforced by law.

## Competing interests

The material for the study was collected as part of an evaluation of the financial subsidies, commissioned and funded by the Swedish Social Insurance Agency and the Ministry of Health and Social Affairs.

## Authors’ contributions

CS wrote most parts of the manuscript, and was responsible for the survey to employers, case interviews and document analyses. AT was responsible for the registry study. CÅ was responsible for the stakeholder interviews. KK was responsible for the survey to OH service providers. All authors contributed to the study design, and all authors read, edited and approved the final manuscript.

## Pre-publication history

The pre-publication history for this paper can be accessed here:

http://www.biomedcentral.com/1471-2458/13/310/prepub
